# Translation, cultural adaptation, and preliminary data evaluation of the Standardized Tool for the Assessment of Bruxism (STAB) and BruxScreen in Turkey

**DOI:** 10.22514/jofph.2026.024

**Published:** 2026-03-12

**Authors:** Berk Bilgen, Mehmet Berk Kaffaf, Pınar Şeşen, Sina Saygılı, Ayşenur Özcan-Sezgin, Süleyman Çağatay Dayan, Hanefi Kurt, Olcay Şakar, Frank Lobbezoo, Daniele Manfredini

**Affiliations:** ^1^Department of Prosthodontics, Faculty of Dentistry, Istanbul University, 34116 Istanbul, Türkiye; ^2^Department of Prosthodontic, Faculty of Dentistry, Istanbul Kent University, 34433 Istanbul, Türkiye; ^3^Department of Dental Services, Vocational School of Health Services, Istanbul Cerrahpaşa University-Cerrahpasa, 34098 Istanbul, Türkiye; ^4^Vocational School of Health Services, Istanbul Bilgi University, 34387 Istanbul, Türkiye; ^5^Department of Orofacial Pain and Dysfunction, Academic Centre for Dentistry Amsterdam (ACTA), University of Amsterdam and Vrije Universiteit, 1081 LA Amsterdam, The Netherlands; ^6^Department of Orofacial Pain and Jaw Function, Faculty of Odontology, Malmö University, 205 06 Malmö, Sweden; ^7^Department of Medical Biotechnologies, School of Dentistry, University of Siena, 53100 Siena, Italy

**Keywords:** STAB, BruxScreen, Bruxism, Bruxism assessment, Bruxism tool, Translation, Cultural adaptation

## Abstract

**Background**: The Standardized Tool for the Assessment of Bruxism (STAB) and Bruxism Screener (BruxScreen) are instruments developed to support the structured evaluation of bruxism across clinical and research settings. For effective use in different populations, translation, cultural adaptation, and preliminary data collection are essential. This study aimed to translate the STAB and BruxScreen into Turkish and evaluate their comprehensibility, feasibility, acceptability, and preliminary clinical applicability. **Methods**: Translation and cultural adaptation of both instruments into Turkish were performed according to original developers’ guidelines. A panel of 12 experts in prosthodontics and/or orofacial pain supervised the process. Independent forward and backward translations were conducted and pilot-tested using the “Three-Step Test Interview” among patients, dentists, and dental students. Additionally, preliminary data were collected using selected components of both instruments to explore their clinical utility. **Results**: Both instruments were translated and culturally adapted. Pilot testing confirmed the face validity and demonstrated high levels of comprehensibility, feasibility, and acceptability across participant groups. Preliminary data collected from patients supported the instruments’ applicability and initial clinical utility within the Turkish population. **Conclusions**: The Turkish versions of both instruments appear to be valid and feasible tools for standardized bruxism assessment. The observed alignment between the outcomes of both instruments underscores their complementary nature and supports their combined use. Their integration may enhance the multidimensional evaluation of bruxism and contribute to international efforts aimed at refining and harmonizing assessment protocols across populations.

## 1. Introduction

The definitions, etiology, and management of bruxism remain widely studied and up-to-date topics [1, 2, 3, 4, 5]. In parallel, expert consensus meetings and recent research have substantially reshaped the conceptualization of bruxism [6, 7, 8, 9]. The most recent consensus statement [8] revised the core definition of bruxism and synthesized ongoing debates into a structured research roadmap aimed at guiding future clinical and academic efforts. The definition of bruxism was revised to become more inclusive and clearly structured. The updated definition emphasizes the behavioral nature of bruxism and acknowledges its association with a broad spectrum of health conditions. Two distinct circadian manifestations are distinguished: sleep bruxism and awake bruxism [6, 7, 8]. This evolving perspective reflects a shift from viewing bruxism as a pathological condition to recognizing it as a motor behavior that may be linked to various health outcomes depending on individual risk factors and clinical context [1, 2, 3].

The long-standing classification system of “possible”, “probable” and “definite” bruxism has been modified to subject-based, clinically based, and device-based, respectively, as concerns about its clinical validity and practical relevance has grown [6, 7, 8, 9, 10]. Bruxism is now increasingly conceptualized not as a dichotomous condition to be diagnosed or excluded, but as a behavior that exists along a continuum, potentially exerting both protective and harmful effects depending on the individual context. In response, recent expert consensus has recommended abandoning rigid diagnostic cut-offs in favor of multidimensional assessment frameworks that capture the complexity and variability of bruxism. This includes considering various methodological sources—such as self-report, clinical examination, and device-based monitoring. Such an approach acknowledges that different assessment tools provide different, yet equally valuable, information about bruxism-related jaw-muscle activity. Accordingly, the development of standardized instruments capable of capturing the multifaceted nature of bruxism has become increasingly important [1, 8, 11, 12, 13, 14].

The definition and understanding of bruxism have evolved significantly during the past decade [7, 8]. However, outdated knowledge persists among dental practitioners. It has been shown that misconceptions about the etiology and management of bruxism remain widespread. Surveys consistently show that a considerable proportion of dentists and dental students continue to rely on traditional occlusal or mechanical explanations, reflecting limited awareness of the current biopsychosocial framework and modern diagnostic standards. Furthermore, many dental curricula still fall short of incorporating updated concepts of bruxism, leading to persistent diagnostic uncertainty [15, 16, 17, 18]. These findings underscore the need for standardized and evidence-based assessment instruments that can support the accurate identification and multidimensional evaluation of bruxism. The cross-cultural adaptation of the Standardized Tool for the Assessment of Bruxism (STAB) and Bruxism Screener (BruxScreen) represents a crucial step toward enhancing conceptual clarity and promoting consistency in bruxism assessment worldwide [3, 11].

To meet this need, an international panel of experts developed two complementary tools: the STAB and the BruxScreen, each serving a different purpose in clinical and research settings [2, 13]. While STAB offers a comprehensive, multidimensional assessment framework, BruxScreen is intended as a brief screening tool requiring fewer items [14]. The STAB is a multiaxial instrument designed to facilitate the structured assessment of bruxism, including its presence and status, potential etiological and associated factors, comorbidities, and clinical consequences. By integrating multiple domains, it offers a comprehensive approach suitable for both clinical application and research purposes [2, 3, 11]. The form consists of two main axes: Axis-A focuses on determining bruxism status and consequences through self-reported, clinical, and instrumental assessments, while Axis-B addresses potential contributing and associated factors based on self-report [2]. A crucial aspect of understanding the concept behind STAB is recognizing that it is not intended to be used in its entirety in all cases. This flexible structure combines validated tools with newly developed components, enabling a multidimensional, yet targeted approach, to bruxism assessment [1, 2, 3].

The BruxScreen, by contrast, prioritizes brevity and clinical accessibility. It was developed as a concise and user-friendly tool to facilitate the rapid identification of both sleep and awake bruxism in routine dental practice and research studies. It consists of two main parts: a self-report section evaluating bruxism behaviors and related symptoms, and a brief clinical section assessing observable signs, such as muscle hypertrophy and dental wear. Unlike the multidimensional STAB, it serves as a screening instrument designed to signal individuals who may require a more comprehensive evaluation using the STAB. From a conceptual standpoint, the BruxScreen aligns with the overarching rationale of the STAB, yet distinct in scope and purpose, as it enables the early identification of potential bruxism cases. It ensures that no patient remains unrecognized in daily clinical practice [13, 14].

Since their development, the STAB and BruxScreen have gained international attention and have been translated into several languages for preliminary use. However, in Turkey, despite the growing clinical awareness of bruxism, validated tools for systematic and standardized assessment have not yet been broadly adopted. A unified assessment strategy is essential to ensure consistency in clinical decision-making and to improve the comparability of data across different populations [2, 7, 12]. To support global applicability, both tools have undergone cultural and linguistic adaptation into several languages through standardized translation protocols, ensuring their conceptual integrity across diverse settings [1, 19, 20].

Accordingly, the present study aimed to translate both the STAB and the BruxScreen form into Turkish following established guidelines [1] and to evaluate their comprehensibility, feasibility, and acceptability through structured pilot testing involving Turkish patients, dentists, and dental students. Furthermore, another aim was to collect preliminary data using STAB and BruxScreen forms, including the frequency of sleep and awake bruxism behaviors, self-reported orofacial complaints/temporomandibular disorder (TMD)-related pain and psychological distress levels, and thus provide insight into the clinical utility of the adapted tools and support their applicability in Turkish-speaking populations.

## 2. Materials and methods

### 2.1 Translation process

The English original versions of both STAB [2] and BruxScreen [13] were translated into Turkish in accordance with the designated template following the necessary permissions obtained through a translation proposal submitted. The cultural adaptation and pilot testing were approved by the Clinical Research Ethics Committee of Istanbul University Faculty of Dentistry under protocol number 2024/46-Rev-1. The process was carried out in accordance with the 12-step guideline provided by the original form developers [19]. The translation team consisted of 12 dentists, including one project leader, three study coordinators (one of whom also served as the team leader), two forward translators, two backward translators, and five expert panelists. All team members met the qualifications required for their roles. The roles and qualifications of the project team are detailed in Table 1.

**Table 1.** Overview of the project members, roles and qualifications.

Project Member	Type of Member	Role(s)	Qualification
Olcay Şakar	Project leader	- Review and approval of translation teams - Review and approval of final version of the form	- Professor and PhD in prosthodontics - Bruxism and temporomandibular disorders expert - Has multiple first-author international publications on bruxism and temporomandibular disorders
Hanefi Kurt	Study coordinator 1	- Synthesis of the two forward translations	- Professor and PhD in prosthodontics - Bruxism and temporomandibular disorders expert - Has multiple first-author international publications on bruxism and temporomandibular disorders - Proficient in English
Berk Bilgen	Team leader and Study coordinator 2	- Synthesis of the two forward translations - Overall responsibility for the project, ensuring progress of the project	- PhD in prosthodontics - Has multiple first-author international publications on bruxism and temporomandibular disorders - Proficient in English
Süleyman Çağatay Dayan	Study coordinator 3	- Identify discrepancies between the source document and common back translation	- Proficient in English - PhD and Assoc. Prof. in prosthodontics - Bruxism and temporomandibular disorders expert
Onur Geçkili	Forward Translator 1	- Translate the original form into Turkish	- Professor and PhD in prosthodontics - Proficient in English
Enes Akpınar	Forward translator 2	- Translate the original form into Turkish	- Specialty trainee in prosthodontics - Proficient in English and target language
Mehmet Berk Kaffaf	Back-translator 1	- Translate the common Turkish translation into the source language	- PhD in prosthodontics - Proficient in English
Canan Bural Alan	Back-translator 2	- Translate the common Turkish translation into the source language	- Professor and PhD in prosthodontics - Proficient in English
Sina Saygılı	Expert Panelist 1	- Review the resulting final translation focusing on semantic, idiomatic, experiential and conceptual equivalencies	- PhD in prosthodontics - Proficient in English and target language
Tonguç Sülün	Expert Panelist 2	- Review the resulting final translation focusing on semantic, idiomatic, experiential and conceptual equivalencies	- Professor and PhD in prosthodontics - Proficient in the target language - Has multiple first and last author international publications on bruxism and temporomandibular disorders - Bruxism and temporomandibular disorders expert
Pınar Şeşen	Expert Panelist 3	- Review the resulting final translation focusing on semantic, idiomatic, experiential and conceptual equivalencies - Representative of the intended study sample	- PhD in prosthodontics - Proficient in English and the target language
Ayşenur Özcan	Expert Panelist 4	- Review the resulting final translation focusing on semantic, idiomatic, experiential and conceptual equivalencies - Representative of the intended study sample	- Proficient in the target language - PhD student in prosthodontics
Afra Avar	Expert Panelist 5	- Review the resulting final translation focusing on semantic, idiomatic, experiential and conceptual equivalencies - Representative of the intended study sample	- PhD student in prosthodontics

The translation process started with two forward translators, each proficient in both the source and target languages, independently translating the original form into Turkish. A synthesis of these two forward translations was then created through a consensus reached by these two researchers and the first study coordinator. This common forward translation was subsequently back-translated into English by two independent back-translators who were bilingual and one of them was knowledgeable in orofacial pain and bruxism. Finally, a consensus among back-translators and the second study coordinator was reached to create the final back-translated version. Subsequently, the third study coordinator, a bilingual bruxism expert, reviewed both versions for discrepancies by comparing them with the original form. Minor discrepancies were observed during this process, primarily involving idiomatic expressions, sociocultural references (*e.g.*, education levels, marital status, alcohol use), and clinical terminologies, such as “sextant”. These discrepancies were discussed among the translation team and resolved through consensus, with the guidance of the expert panel when necessary, prioritizing conceptual and semantic equivalence over literal translation. After consensus was achieved, the ultimate Turkish version was reviewed one last time by the study coordinators and the project leader. The panel consisted of two bruxism experts, two PhD students, and a dentist with native-level proficiency. Upon completion of this review, the translation process was finalized.

### 2.2 Pilot testing

To evaluate the comprehensibility, feasibility, and acceptability of the Turkish version of both forms, as well as to document the experience of both clinicians and patients completing it, on-field pilot testing was conducted in Prosthodontics Departments of two different faculties of dentistry: Istanbul University and Istanbul Kent University. Participant recruitment and data collection were carried out between May 2025 and July 2025. The pilot testing process was carried out on a total of 60 people, in accordance with the criteria and principles established by the authors of the form [1, 19]. The test was conducted on 20 patients, including 14 women and 6 men, aged between 18 and 59 years, with a mean age of 33.6 ± 12.5 years. Twelve participants were referred to the orofacial pain department, while eight sought treatment in general dentistry. The group represented a wide range of educational backgrounds: nine were high-school graduates, six were university graduates, two had completed associate degrees, two had completed compulsory education, and one held a postgraduate qualification. Patients with more than one missing tooth in any quadrant, those who were illiterate, had intellectual disabilities, or had previously participated in surveys related to orofacial pain complaints were excluded from the pilot testing. Each patient was independently evaluated by one dentist and one dental student. All 20 dental students were in their graduation year and had completed most of their clinical and theoretical training, with less than six months remaining until graduation. As part of the curriculum, all students had received formal theoretical education on temporomandibular disorders, orofacial pain, and bruxism. The group of 20 dentists included 8 postgraduate students in prosthodontics with more than three years of clinical experience, 5 prosthodontic specialists who regularly treat patients with orofacial pain and temporomandibular disorders, 4 experts in orofacial pain, 2 periodontologists, and 1 general practitioner. All participating dentists had received postgraduate education in prosthodontics.

All interviews with patients, dental students, and dentists were conducted individually by the expert panelists. Based on the methodology described by De Vet *et al*. [21] the “Three Step Test Interview” method was followed. The primary aim of the pilot test was to evaluate the comprehensibility, feasibility, and acceptability of the Turkish version of the form.

Regarding comprehensibility, all participants were asked to identify any items they found unclear or confusing, whether in terms of wording, explanations, or response options. Participants were encouraged to elaborate on specific items they found ambiguous or difficult to interpret. For feasibility, they were questioned about their ability to complete the form independently, without any assistance, within an adequate time span.

Acceptability was assessed by evaluating the willingness of participants to complete the form. Clinicians and dental students were specifically asked whether they would consider using the form in their future clinical practice, and to justify their responses. Their opinions were also gathered regarding the perceived length of the form and its usefulness in aiding clinical diagnosis. Patients were asked to share their overall impressions, including whether they found the questions relevant to their complaints and whether they encountered repetitive sections.

All feedback obtained during the interviews was documented using standardized feedback sheets and detailed field notes. Short audio recordings were also taken to minimize recall bias and ensure accurate documentation of qualitative feedback; these recordings were used solely for transcription purposes. Each participant’s qualitative remarks, including suggestions for clarification, perceived redundancy, or cultural relevance, were transcribed verbatim by the examiner immediately after each session. Responses were then thematically categorized under comprehensibility, feasibility, and acceptability domains, enabling systematic comparison across patients, students, and dentists. This approach ensured transparent documentation of subjective impressions and supported face validity assessment in alignment with established methodologies for questionnaire translation and pilot testing.

The completion time was objectively measured for each participant using a digital stopwatch. Timing began when the participant started reading the first item and ended upon completion of the last item of each form. This procedure was applied to both patients completing the self-report sections and to clinicians (dentists and students) completing the clinical parts of both forms. This approach allowed a standardized and observer-recorded estimation of form completion time. In line with the procedure adopted in the Italian adaptation study by Colonna *et al*. [1] a 20-minute duration was defined as the acceptable upper limit for the self-reported sections of the STAB, and 15 minutes for its clinical section.

### 2.3 Preliminary data collection and descriptive analysis

Following the completion of the translation and pilot testing procedures, preliminary data were collected from 20 participating patients using selected sections of the Turkish version of the STAB and BruxScreen-Q. These data aimed to evaluate the initial clinical utility of the Turkish version and to explore bruxism-related behaviors among Turkish patients. Specifically, three validated components embedded in the STAB were utilized: the Oral Behavior Checklist (OBC), the TMD Pain Screener, and the Patient Health Questionnaire-4 (PHQ-4). Additionally, all items from the first question of the BruxScreen-Q, which assess both sleep and awake bruxism behaviors, were included in the analysis.

The OBC, integrated across Axis A and Axis B of the STAB, comprises 21 items that collectively assess the frequency of awake and sleep-related oral behaviors. Among these, two items are specifically dedicated to sleep-related oral behaviors, A1.1 (sleep bruxism frequency) and B2.4 (sleep position), while the remaining 19 items reflect awake behaviors. Scores were calculated separately for awake and sleep behaviors and as a combined total, allowing a more detailed evaluation of behavioral patterns [22, 23].

Responses to the first question of the BruxScreen-Q were similarly analyzed to capture both sleep and awake bruxism behaviors [13]. The BruxScreen-Q uses a 0–4 ordinal scale (never–always); as no validated cut-offs exist, responses were interpreted qualitatively. The reported frequencies were documented and subsequently compared to the corresponding scores obtained through the STAB’s OBC to explore the level of agreement between the two instruments.

The TMD Pain Screener, located in Axis A, includes six items scored either 0–2 or 0–1, depending on item format and the PHQ-4, embedded in Axis B, screens for psychological distress through four items related to anxiety and depression. Each item is rated from 0 to 3 [23]. All corresponding cut-off values and clinical interpretation criteria for each instrument are summarized in Table 2.

**Table 2.** Summary of descriptive results and clinical interpretation for STAB and BruxScreen domains.

Instrument	Domain	Mean ± SD	Range	Clinical Interpretation
OBC				
	Awake Behaviors	14.5 ± 5.8	6–35	-
	Sleep Behaviors	3.7 ± 1.1	2–6	-
	Total Score	18.2 ± 9.3	4–50	0–16: normal; 17–24: increased; ≥25: high/TMD risk
BruxScreen-Q				
	Awake Behaviors (1c–1f)	2.7 ± 0.9	1–4	No validated cut-off
	Sleep Behaviors (1a–1b)	2.3 ± 1	0–4	No validated cut-off
TMD Pain Screener	Total Score	1.5 ± 1.2	0–5	≥3: possible TMD pain
PHQ-4	Total Score	1.2 ± 1.7	0–7	0–3: none; ≥3: mild; ≥6: moderate; ≥9: severe distress

*OBC: Oral Behavior Checklist; PHQ-4: Patient Health Questionnaire-4; TMD: Temporomandibular Disorders; BruxScreen: Bruxism Screener; SD: Standard Deviation.*

## 3. Results

The pilot testing was conducted with a total of 60 participants, including 20 patients (14 females, 6 males; mean age: 33.6; age range: 18–59 years—Table 2), 20 dentists (11 females 9 males; mean age: 31; age range: 24–38), and 20 dental students (12 females, 8 males, mean age: 23.9 age range: 23–26 years), with each patient independently evaluated by one dentist and one dental student.

The comprehensibility of the Turkish version of both the STAB and the BruxScreen was generally found to be high across all participant groups. All patients reported that the Turkish versions were clear and understandable, including the language, idiomatic expressions, and phrasing. Six patients noted that completing both forms helped them become more aware of their possible bruxism-related behaviors, such as clenching and chewing habits. Notably, none of the patients reported any difficulties in understanding the BruxScreen form, and several explicitly highlighted its clarity and simplicity. In contrast, comprehension difficulties were observed exclusively in two specific subsections of Axis B of the STAB: *Orofacial Motor Disorders* and the *Autoimmune or Connective Tissue Disorders Screening*. A total of 11 patients indicated that they had difficulty understanding the response options and were therefore unsure how to respond. Of these individuals 63.6% had completed only secondary education or less. Additionally, four participants reported unfamiliarity with the drugs listed in the *Medication *subsection (*e.g.*, benzodiazepines, dopamine antagonists, and selective serotonin reuptake inhibitors), and suggested that the use of common brand names might improve clarity for individuals without a medical background.

Dental students and dentists also evaluated the comprehensibility of both Turkish versions positively. None of the participants in these groups reported any difficulties related to linguistic clarity, and the form was described as clear, coherent, and easy to follow. However, in both forms, two specific components were identified as unfamiliar by the vast majority of the participants: the term “sextant” and tooth wear scoring system. Neither component was understood by any of the dental students or of the 16 dentists.

Apart from the *Tooth Wear* and *Scoring* subsections, left unanswered by some dentists and dental students, all participants were able to complete the Turkish version of the forms independently, without requiring clarification or external assistance. The average completion time for the self-report component of the STAB was 16 minutes and 49 seconds among patients (range: 7:02–40:28). Except for one individual, all patients reported that the form was completed within an acceptable timeframe and that they experienced no difficulties during completion. For the BruxScreen form, patients completed the questionnaire in an average of 3 minutes and 28 seconds (range: 1:32–6:00). Regarding the clinical component of the STAB, the average completion time was 10 minutes and 38 seconds among dental students (range: 6:51–17:13), and 8 minutes and 16 seconds among dentists (range: 5:17–14:44). For the BruxScreen-C (clinical assessment form), average completion times were notably shorter: 2 minutes and 40 seconds for dental students (range: 1:09–6:01) and 1 minute and 51 seconds for dentists (range: 1:03–3:21). In both forms, as expected, dental students required more time than dentists to complete the clinical sections. The shorter completion times for BruxScreen-C were largely attributed to its brief structure and the frequent omission of the sextant and tooth wear scoring sections, which many participants found unfamiliar.

The Turkish versions were generally well accepted by participants across all groups. All patients considered the form acceptable in terms of length and content and none reported discomfort or hesitation during completion. Although, six patients noted that some questions in the STAB form felt repetitive, particularly within the “Awake Bruxism” and “Temporomandibular Disorders” subsections, where similar phrasing was used across multiple items; the vast majority reported that the STAB form allowed them to better express their complaints. Regarding the clinical assessment sections of both forms, dental students and dentists found them generally acceptable. However, preferences differed between groups. Most dental students favored the STAB form, stating that its comprehensive structure allowed for a more thorough examination of the patient. In contrast, many dentists preferred the BruxScreen form, highlighting its shortness and ease of use, which they believed would be less disruptive to clinical workflow. Eighteen out of twenty dental students expressed interest in incorporating the STAB form into future clinical practice and research, appreciating its structured format and detailed content. The remaining two students did not find the form particularly relevant to their clinical focus, citing limited prior exposure to orofacial pain and bruxism. Among dentists, although the majority valued the multidimensional nature of the STAB, eight expressed uncertainty regarding the interpretation of its results and how they could be translated into clinical decision-making.

Descriptive results are presented in Table 2. The mean OBC score for sleep-related behaviors was 3.7 ± 1.1, while the mean score for awake oral behaviors was 14.5 ± 5.8. The mean total OBC score was 18.2 ± 9.3. Eight patients (40%) scored ≥25, exceeding the threshold considered indicative of increased oral behavior frequency and a potential TMD risk. Notably, seven of these individuals also had TMD Pain Screener scores ≥3, supporting the concurrent presence of pain-related risk indicators.

Patient responses to the first item of the BruxScreen-Q showed comparable behavioral patterns. For the two sleep-related items (a and b), 33.4% of patients reported clenching or grinding “frequently” or “always”, while among the four awake-related items (c–f), 45–55% reported engaging in the respective behaviors at least “regularly,” with the tooth clenching being the most prevalent (55.2%). These self-reported frequencies showed moderate correlation with OBC subscores, especially in the awake domain.

The mean TMD Pain Screener score across all participants was 1.5. Among the patients referred with orofacial pain, the mean score was 2.33, whereas those attending for general dentistry reasons had a substantially lower average of 0.25. The mean PHQ-4 score was 1.2. Twelve patients demonstrated higher scores (mean: 2.26) compared with the remaining eight (mean: 0.25), consistent with the broader clinical profiles. However, none of the participants exceeded the established cut-offs for moderate or severe distress.

## 4. Discussion

This study aimed to translate and culturally adapt both the STAB and BruxScreen forms into Turkish and to assess their comprehensibility, feasibility, and acceptability through structured pilot testing among patients, dentists and dental students. As the international application of both instruments expands, they hold strong potential to standardize the assessment of bruxism, thereby supporting consistent and comparable data collection in both research and clinical contexts. By integrating subjective, clinical, and instrumental domains, both instruments provide a robust framework for evidence-based evaluation of bruxism-related phenomena [2, 3, 11]. Furthermore, the preliminary data collected using selected sections of the Turkish version of both forms, covering the bruxism behavior frequency, orofacial complaints, TMD-related pain, and psychological distress, offered initial insights into the tools’ clinical utility and applicability within Turkey.

The present findings reinforce the importance of updating clinical and educational practices in bruxism assessment [15]. A recent study conducted in Turkey similarly demonstrated that dental students, despite moderate theoretical awareness of bruxism, often lack a comprehensive understanding of its multifactorial nature and show low confidence in its clinical evaluation [18]. Therefore, the translation and cultural adaptation of the STAB and BruxScreen into Turkish hold importance, as they can help integrate evidence-based knowledge into clinical and educational settings, enhance diagnostic reliability, and foster a more standardized approach to bruxism assessment. In addition, recent evidence suggests that different bruxism phenotypes may lead to distinct clinical consequences, further underlining the need for standardized assessment. Traditional approaches that viewed bruxism as a single condition often overlooked the variability between clenching and grinding behaviors, or between sleep and awake manifestations. It was demonstrated that pain localization patterns significantly differ across bruxism types [24], highlighting the clinical relevance of differentiating between bruxism phenotypes and supporting the adoption of multidimensional tools, such as the STAB and BruxScreen, which are specifically designed to capture this behavioral diversity and its potential consequences.

The “Three-Step Test Interview” method, incorporating both “probing” and “think-aloud” techniques was used to assess not only whether participants understood the questions, but also how they interpreted and processed the content within their individual mindsets. This method facilitated the evaluation of face validity by exploring how the intended meaning of the forms aligned with individual participant perspectives [21].

Qualitative feedback from participants and an expert panel confirmed the conceptual equivalence of the Turkish version and supported its face validity. However, both the STAB and BruxScreen remain under refinement, with ongoing international field testing informing structural updates and cross-cultural improvements. A key concern raised—particularly by dentists—was the lack of a practical framework for interpreting results, specifically regarding scoring and diagnostic thresholds. This highlights the need to develop structured interpretation guidelines, including clinical cut-offs and decision-making algorithms, to enhance usability in daily practice [3, 11, 13].

While adapting a professional tool for use in different languages, it is essential to consider the cultural norms, lifestyle, and contextual characteristics. These cultural considerations may require minor modifications to ensure that the translated version remains conceptually accurate and easily understood by the intended users [19, 20, 25]. During the translation process, several minor discrepancies were identified and resolved through consensus to ensure cultural and conceptual alignment. For instance, the “education level” item in the STAB was modified by replacing the “certificate program” option with “associate degree”, which better reflects the Turkish education system, where short-term certification programs are not formally standardized. Similarly, certain response options related to frequency descriptors (*e.g.*, “sometime” and “regularly”) were discussed in depth, as their semantic distinction was perceived as subtle in Turkish. The final decision was to retain the original phrasing to maintain consistency with the source version while documenting this potential overlap for future psychometric evaluation. The clinical term “sextant” also posed a translation challenge due to its limited use in Turkish dental education; in Turkey, dental examinations are typically structured by quadrants rather than sextants, and the Tooth Wear Evaluation System (TWES) [26], from which this terminology originates, is not routinely utilized in clinical practice. To address this, it was decided to retain the term but supplement it with an explanatory illustration to enhance clarity and ensure accurate comprehension. Beyond these minor adjustments, qualitative feedback from participants further supported the conceptual equivalence and face validity of the Turkish versions. While some elderly patients found the distinction between “divorced” and “separated” unclear, the original categories were retained due to adequate overall comprehension. A small number of patients expressed discomfort with items on alcohol or substance use, yet most found them acceptable, warranting no further revision.

Pilot testing later confirmed that certain self-report frequency descriptors were occasionally interpreted inconsistently, particularly in the items assessing bruxism-related behavior. Participants sometimes struggled to distinguish between “sometimes” and “regularly”, mirroring the concerns identified during translation. This observation is consistent with previous findings by Grossi and Filho [27], who noted that verbal rating scales may lead to inconsistent interpretations, particularly for items measuring behavioural frequency. In response to such concerns, the developers of the BruxScreen emphasized that these qualitative descriptors were deliberately chosen for their clinical convenience, arguing that patients often struggle more when asked to recall specific numerical frequencies [14]. Nonetheless, several participants in the Turkish pilot suggested that using numerical frequency ranges might make interpretation more straightforward and reduce misunderstandings arising from cultural or educational differences.

Regarding the comprehensibility, nearly all items were clearly understood. Among patients, no major difficulties were encountered concerning the linguistic clarity of the translated version. The only challenge observed among patients involved questions related to medical history in the STAB, likely due to unfamiliarity with medical terminology and lower health literacy levels, rather than flaws in translation [28]. This interpretation is further supported by the observation that the majority of these individuals were only high school graduates or less, suggesting that educational background may have contributed to the observed challenges. Several participants recommended adding an “I don’t know” option, particularly for items concerning systemic diseases or medication groups. In contrast, the BruxScreen was uniformly described as easy to understand, concise, and coherent.

Feasibility was assessed through form completion time and overall manageability. In the Italian STAB validation, a completion time of 20 minutes for the self-reported section and 15 minutes for the clinical section was defined as an acceptable threshold [1]. In the present study, the average completion times observed for remained below these thresholds, supporting the practical applicability of the form. However, some participants skipped or minimally engaged with the tooth wear and sextant-based evaluation sections, which may have resulted in shorter completion times. Still, several dentists perceived the STAB as relatively long. The highest variation in completion time was observed among patients, likely reflecting individual differences in reading speed, familiarity with medical terminology, and overall health literacy [28]. Nonetheless, all patients completed the form within a clinically acceptable timeframe, further supporting the feasibility. To date, no prior study has assessed the feasibility or completion time of the BruxScreen form. This study, thus, represents the first empirical investigation into its practical application. As expected, the BruxScreen required considerably less time to complete than the STAB. As particularly with the STAB, dental students required more time to complete the clinical section than dentists, which may be attributed to their limited clinical experience and slower interpretation of diagnostic parameters.

The overall acceptability of the instruments was high across all participant groups. Patients found both tools helpful for expressing their complaints. Dentists favored the brief, practical nature of the BruxScreen for routine use, while dental students preferred the structured and comprehensive STAB—likely influenced by their training focus. These patterns suggest that the two instruments are conceptually aligned, yet serve distinct purposes: the BruxScreen offers a rapid screening framework for everyday clinical use, whereas the STAB provides a multidimensional evaluation suitable for detailed clinical and research assessment [2, 13].

To further explore the clinical applicability of the Turkish adaptation, descriptive data were collected from key subsections of both STAB and BruxScreen, which are known to reflect bruxism and TMD risk indicators [2, 3, 23]. Collectively, these results capture behavioral, symptomatic, and psychological domains, aligning with the multifactorial nature of bruxism, while offering preliminary support for the clinical relevance and differential sensitivity of the assessment tools. The elevated OBC and TMD Pain Screener scores among patients referred for orofacial pain suggest a convergence of behavioral and pain-related risk factors, in line with earlier studies reporting comorbid patterns between high oral parafunction and TMD symptoms [23, 29]. Similarly, although none of the patients in this pilot met the criteria for moderate or severe psychological distress, PHQ-4 scores varied and indicated that emotional factors may act as contextual contributors to bruxism, consistent with earlier literature emphasizing subclinical emotional distress as a relevant perpetuating factor [23, 28, 30].

Overall, this pilot study revealed coherent patterns across behavioral, pain-related, and psychological domains. These preliminary findings suggest that increased oral behavior frequency, as indicated by the OBC, tends to co-occur with higher pain screener scores and mild emotional distress, supporting the multifactorial conceptualization of bruxism and its association with biopsychosocial risk factors. Such observations underscore the potential clinical relevance of integrating multidomain screening tools to capture both behavioral and psychosocial correlates of bruxism within routine diagnostic workflows [10].

Beyond STAB results, the integration of sleep and awake bruxism data from the first item of the BruxScreen-Q provided an additional behavioral insight. A substantial proportion of patients reported frequent or consistent bruxism behaviors during both sleep and wakefulness. Importantly, patients who indicated frequent awake bruxism behaviors in the BruxScreen-Q tended to show correspondingly higher awake-related OBC scores. This alignment between two independently developed tools supports their potential convergence and construct validity. In particular, it highlights the convergent and sequential utility of employing both instruments to capture the temporally distinct expressions of bruxism [4, 6]. Nevertheless, this moderate alignment should be interpreted with caution. The small sample size and descriptive nature of the present pilot may have amplified the strength of this association [21]. Thus, while the observed correspondence between the OBC and BruxScreen-Q results provides a preliminary indication of convergent validity, it cannot be taken as definitive evidence of construct equivalence. Potential differences between the two instruments should not be regarded as an inconsistency, since the two forms were not designed as a full and short version of the same instrument. Rather, they represent complementary tools addressing different levels of clinical assessment. The STAB offers a multidimensional evaluation framework integrating subjective, clinical, and instrumental domains, whereas the BruxScreen serves as a concise screening tool for rapid identification of bruxism-related behaviors [2, 3, 13]. Consequently, a partial overlap between their behavioral domains is theoretically expected and reflects their distinct, yet compatible, scopes of application.

While this study did not include statistical testing due to its limited sample size, the descriptive analyses provided preliminary support for the clinical sensitivity of the Turkish versions, showing promising differentiation across behavioral, pain-related, and psychological domains. However, the small number of participants represents an inherent limitation, restricting the generalizability of the findings and precluding any inferential statistical testing. The pilot nature of the study also implies that the observed tendencies should be interpreted with caution, as they primarily serve to inform feasibility, linguistic validity, and preliminary clinical insights rather than to establish definitive associations. Future research involving larger and more diverse clinical populations is needed to validate these preliminary observations, determine psychometric robustness and establish clinically meaningful thresholds for Turkish populations. These efforts will be critical in advancing the standardization of bruxism assessment tools for international clinical and research use.

## 5. Conclusions

The findings of this pilot study support the cultural and linguistic appropriateness of the Turkish versions of both the STAB and the BruxScreen. The translated instruments were generally perceived as comprehensible, feasible, and acceptable across all participant groups. Feedback from patients, dental students, and clinicians confirmed their face validity and highlighted their perceived utility in the assessment of bruxism-related features. Notably, the few concerns raised during pilot testing were not related to the translation process itself, but rather to broader challenges regarding the integration of the original forms into routine clinical workflows, particularly the interpretation of scoring outputs and the absence of standardized diagnostic cut-off values. Nevertheless, these findings should be interpreted within the limitations of a small pilot cohort and a descriptive analytical design. Further large-scale validation studies are required to confirm these preliminary results and to refine the clinical applicability of both instruments in Turkish-speaking populations.
